# “Fatal Gastrointestinal and Peritoneal Ischemic Disease” of Unknown Cause at Arba Minch Hospital, Southern Ethiopia

**DOI:** 10.1155/2018/6598960

**Published:** 2018-10-23

**Authors:** Jilcha Diribi Feyisa, Melka Kenea, Efrem Gashaw, Eskezyiaw Agedew Getahun, Barry Leon Hicks, Hailemichael Desalegn

**Affiliations:** ^1^Department of Internal Medicine, Saint Paul's Hospital Millennium Medical College, Ethiopia; ^2^Department of Surgery, Arba Minch General Hospital, Ethiopia; ^3^Department of Public Health, College of Health Sciences, Arba Minch University, Ethiopia; ^4^Department of Surgery, Arba Minch University, Ethiopia

## Abstract

Gastrointestinal and peritoneal ischemic disease due to unknown etiology present with intestinal obstruction and/or peritonitis otherwise in healthy patient emerged as fatal disease at Arba Minch General Hospital. This disorder was diagnosed based on intraoperative finding. Clinical presentation and natural history of disease progression were similar. It is estimated that about 6–10 lives are being claimed each year at Arba Minch Hospital with this disease of unidentified cause accounting for the largest figure of surgical department. Here we report case analysis and literature review illustrating clinical presentation, workup, preoperative diagnosis, intraoperative diagnosis, and final outcome of fatal gastrointestinal and peritoneal ischemic disease.

## 1. Introduction

Fatal gastrointestinal and peritoneal ischemic disease (FGPID) is a pathology which describes ischemic changes to intraperitoneal organs including bowel, peritoneum, omentum, gallbladder, mesentery, and mesenteric lymph nodes. Although this disease is acknowledged as emerging problem at Arba Minch General Hospital (AMGH) years ago, there was no study so far in this hospital to identify cause and possible factors behind, whereas the mortality is high and continuing. This is clearly reflected by outnumbered death reports during surgical morning session and monthly reports. Besides, there are no in depth clinical evaluation, investigation, and recorded data of those patients apart from having the routines. Therefore, the definite pathology, possible cause, natural history, and immediate cause of death remain obscure. The blindly tried options of managements are unsuccessful so far.

We could not identify similar case reports, but entire and localized bowel ischemia can occur due to vascular and nonvascular causes identified due to eosinophilic gastroenteritis and mesenteric vascular disorders [1, 2]. Ischemia of intraperitoneal organs with obstruction feature are identified in these reports specifically with eosinophilic gastroenteritis (EGE) and ischemic bowel disease/mesenteric ischemia (MI) [3–5].

## 2. Case Presentation and Analysis

In this report we analyzed medical records of 8 consecutive patients who were operated and found to have ischemia of peritoneum and the whole bowel from April 1/2015 to December 1/2016 in AMGH, southern Ethiopia. A retrospective analysis of cases and literature review have been done. Data was collected using structured data sheet. The results were annualized manually and descriptive statics were used.

**Figure 1:**
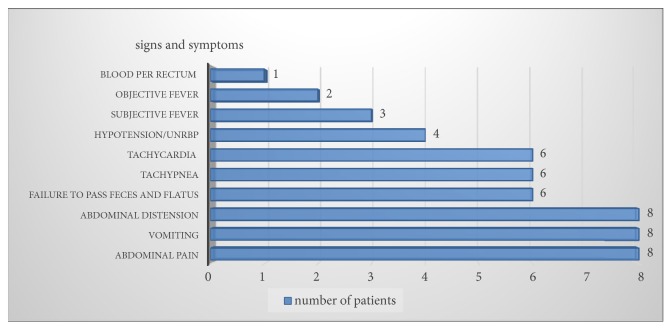
Clinical symptoms and signs on presentation of patients with fatal gastrointestinal and peritoneal ischemic disease discussed in this case report. UNRBP: unrecordable blood pressure.

Among 8 patients analyzed with gastrointestinal and peritoneal ischemic disease of unknown cause in Arba Minch General Hospital, 6 were females and 2 males, with mean age of 26 years ±12.54 and SD range of 11-45 years. 4 of the patients are from Chencha woreda while the rest are from Bonke woreda. All patients were presented as acute abdomen. Abdominal pain, vomiting, and abdominal distension were presenting symptoms in all cases (Figure 1). Six of patients had failure to pass feces and flatus. Three of the cases presented with hypovolemic shock. Six of them had tachycardia and all were tachypneic, with average pulse rate of 119 ±25.83 bit/minute and average respiratory rate of 36± 8.25 breath/minute. Only two of the cases had objective fever with axillary temperature of >38°C and average temperature of 37.47°C±1.2 SD on presentation.

**Figure 2:**
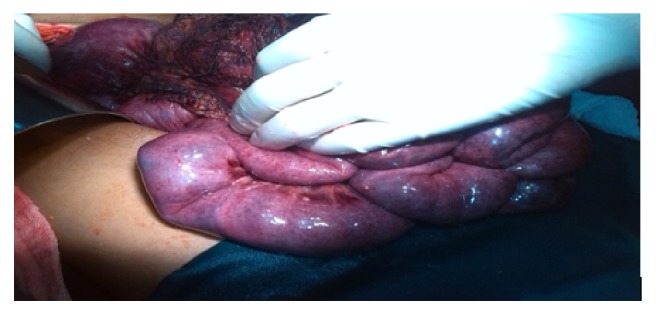
Gross specimen showing patchy ischemia of small intestine, peritoneum, and omentum from patient having gastrointestinal and peritoneal ischemic disease.

The preoperative clinical diagnosis in six of the cases was small bowel obstruction with generalized peritonitis. Three of them were presumed to have generalized peritonitis, one acute pancreatitis, and one suspected to have mesenteric ischemia. Two of the cases were consulted to obstetric side for possible ruptured ovarian cyst. The intraoperative finding in all eight cases, however, confirmed gastrointestinal and peritoneal ischemic disease with no cause identified. All patients had ischemic changes which involved the whole bowel (stomach, small bowel, large bowel to rectum), peritoneum, and omentum (Figure 2). Pancreas condition was normal except one case which got edematous. In addition all cases have hemorrhagic ascites. Six patients were found to have abdominal wall involvement while gallbladder was involved in one case. Mesenteric lymphadenopathy (LAP) was observed only in 2 cases. Mesenteric arteries are found pulsating in all of the cases.

Regarding outcome after operation, five of the cases died in hospital within 2 hours up to eighth postoperative day's intervals. One patient was self-discharged against medical advice as she was getting deteriorated. She did not revisited hospital and hence her final outcome was not traced. One patient was improved and discharged on 21^st^ postoperative day. She was appointed to surgical referral clinic but she did not appear on her appointment date. Her case was so not confirmed as she was not traced for her final outcome (Table 1).

## 3. Discussion

The present report addressed ischemic changes to entire bowel, peritoneum, and other intraperitoneal viscera's with ileus as remarking presentation. There have been reports on intestinal and/or peritoneal ischemic changes with different clinical presentation from different corners of the world specifically from far east countries even if hardly available with similar presentation [1–6]. There is a paucity of literature in our country, Ethiopia, regarding ischemic disease involving intraperitoneal viscera. Oral based discussion made among surgical seniors from Black Lion, Jimma, Gondar, Hawassa, and Arba Minch University Hospitals however indicated that a number of patients were seen in Jimma hospital Having ischemia of the whole bowel (personal communication, Seifu, MD, 2015, AMGH). Interpersonal communication made with medical practitioner from Mizan Tepi Hospital also revealed the presence of similar cases diagnosed on the basis of intraoperative finding (personal communication, Ruth Shimelis, MD, November 2016, SPHMMC).

The result of the analysis showed invariable ischemic changes of stomach, small bowel, large bowel, peritoneum, and omentum. Matsushita M et al. reported eosinophilic gastroenteritis involving the entire digestive tract [1]. The result of our study indicated that six of the patients were presumed to have intestinal obstruction. A case series report from Mangalore, South India, of four patients who presented with intestinal obstruction showed a similar clinical presentation to our cases. Histopathological report of those cases confirmed EGE in all cases [3]. All presented with abdominal pain, vomiting, weight loss, diarrhea, and abdominal distension which are the major presenting symptoms of EGE in descending order as per a study done in USA by Talley on clinicopathological aspect of EGE [3, 7]. Hemorrhagic ascites were found in all of the case. As per Klein classification of EGE, the muscular layer involvement was found to be present with intestinal obstruction while serosal type is studied as the cause for ascites [8]. Female predominance is found in the same report (three females and one male) though other reports on EGE showed male predominance [3, 7]. EGE commonly occurs in children. Mean age at diagnosis is third to fifth decade, commonly in fifth decade which is closely parallel age group of our case [7]. Clinical misdiagnosis is common in EGE especially when it is present with obstructive feature. In the case report from India, patients are clinically misdiagnosed as Intestinal Tuberculosis, volvulus, neoplasm, and other uncertainty for obstruction [3]. This is because diagnosis of EGE is challenging and one of the exclusion pathologies [8]. This might be similar to our cases where there is clinical misdiagnosis though the actual pathology is yet not confirmed. EGE was repeatedly reported as allergic condition in more than half of the cases [9]. The seasonality and area specific nature of our cases may so suggest its possibility. It is also reported in connection with parasitic infection [10]. Beside Ascariasis found intraoperatively in one of our cases, the burden of infectious disease in the region which can have systemic presentation involving those anatomical parts needs high consideration in this regard raising the possibility.

The other reasonable pathology mentioned in literature resulting in ischemic change to bowel is obstruction of mesenteric arteries or systemic hypoperfusion termed as mesenteric ischemia [2, 6, 11]. Consistent with our case, it is repeatedly reported as fatal condition necessitating immediate medical and surgical intervention. A clinical series done over the last 15 years summarizing the possible outcome of acute type mesenteric ischemia showed a mortality rate of 70% with a range of 59%-93%. Clinical analysis done 70 years earlier than this report also showed similar figure [11]. It is comparable to 75% fatality of our case. Mesenteric ischemia is reported to affect every segment of gastrointestinal tract. Many of case reports are however on isolated small bowel or colonic ischemia since multiple collateral vessels are available in upper segment. Ischemic colitis is commonly reported in association with mesenteric vascular disease [1, 2, 12]. A case report from Staten Island University Hospital, Staten Island, NY, USA, explains an ischemic bowel injury which occurred following angiography [6]. It needs obstruction of abdominal aorta, small mesenteric arterial occlusion, or nonocclusive type mesenteric ischemia to result in involvement of the whole bowel and other intraperitoneal viscera [13]. This contradicts our finding of whole bowel involvement in all cases. Intraoperatively identified pulsatile mesenteric arteries of patients in our case also question the possibility of mesenteric ischemia. Still it cannot be ruled out with the possibility of small penetrating arterial occlusion or nonocclusive type of mesenteric ischemia. Similar to our cases abdominal pain is the usual manifestation of mesenteric ischemia mimicking peritonitis. It outranges abdominal finding raising high clinical suspicion of the case. According to a study done in series of 58 cases, abdominal pain was present in 95% of cases while metabolic acidosis (44%), nausea (44%), vomiting (35%), diarrhea (35%), tachycardia (33%), blood per rectum (16%), and constipation (7%) were also present [11, 14]. Except diarrhea and unconfirmed metabolic acidosis, all were the presenting features of our cases.

We could not find literature which explains the involvement of gall bladder, omentum, and abdominal wall with this listed clinical presentation found in our case.

## 4. Limitations

Incomplete recording of medical records, limited laboratory investigations done, unavailability of imaging modalities, failure to collect histopathological result sent as the fatal nature of disease resulting in death before bringing to hospital, difficulty of tracing family back due to lack of precise address on cards were the limitations faced.

## 5. Conclusion

The involvement of the whole bowel with peritoneum, omentum, abdominal wall, and gall bladder in some cases without selection of arteries, presence of mesenteric pulse intraoperatively, evidences high possibility of nonvascular disease. Absence of cardiac and renal comorbidities also makes nonocclusive disease unlikely. Higher incidence observed among third to fifth decade (25.75 years ±12.54 SD) having age range of 11-45 years with inclusion of children as well, higher incidence in female, patchy nature of ischemia, presence of hemorrhagic fluid, acute onset of disease, nonmechanical nature of intestinal obstruction, and area and season specific nature of the disease suggest the possibility of exposure to food allergens or nutritional poisoning, environmental toxins, or allergens. The presence of tachypnea in all of patients might also strengthen the possibility of allergen induced hypersensitivity. The possibility of infectious cause and autoimmune or neoplastic causes is still not ruled out.

## Figures and Tables

**Table 1:** Postoperative outcome of patients diagnosed with FGPD of unknown cause.

Patient	Outcome	Date of death/discharge (post-operative)	Condition on discharge (if discharged)	Condition after discharge (if discharged)
Patient 1	Death	4^th^		

Patient 2	Death	8.5 hours		

Patient 3	Discharge	21^st^	Improved, appointed to SRC	Not revisited, not traced

Patient 4	Death	2 hours		

Patient 5	Self-discharge	8^th^	Deteriorated	Not revisited, not traced

Patient 6	Death	6^th^		

Patient 7	Death	In Recovery Room		

Patient 8	Death	3^rd^		

SRC: surgical referral clinic.

## Data Availability

The data sets that were analyzed in this case report are available from the corresponding author on reasonable request.

## Additional Points

*Recommendation*. We recommend the need for further research to identify the possible cause of this fatal illness. Based on different literature sources and experience, the nature of intraoperative features affecting specific communities in a seasonal pattern needs to have urgent multidisciplinary approach from clinicians, epidemiologists, and pathologists to identify the cause of this fatal disease and recommend effective measures.
